# Outcomes of First-Line PARP Inhibitor Therapy in Ovarian Cancer: A Multicenter Retrospective Analysis

**DOI:** 10.3390/jcm15041657

**Published:** 2026-02-22

**Authors:** Baris Koksal, Hasan Cagri Yildirim, Deniz Can Guven, Fatih Kose, Gorkem Koymen, Ozlem Ozdemir, Ugur Ozberk, Efnan Algin, Murad Guliyev, Nebi Serkan Demirci, Ozkan Alan, Ahmet Baklaci, Bilgin Demir, Ozlem Topkaya, Necdet Uskent, Kadriye Bir Yucel, Orhun Akdogan, Bahadir Koylu, Fatih Selcukbiricik, Irem Bilgetekin, Kaan Helvaci, Ahmet Unal, Huseyin Salih Semiz, Teoman Sakalar, Nadiye Sever, Ceren Mordag Cicek, Gamze Gokoz Dogu, Sedat Biter, Ertugrul Bayram, Canan Yildiz, Hacer Demir, Ismail Bayrakci, Bulent Erdogan, Mehmet Emin Kalender, Halil Goksel Guzel, Banu Ozturk, Berkan Karabuga, Ozturk Ates, Emine Bihter Cetin, Mustafa Sahbazlar, Ali Inal, Veli Sunar, Fatma Bugdayci Basal, Esra Asik, Atila Yildirim, Nejat Emre Oksuz, Yuksel Urun, Erdinc Nayir, Sema Turker, Sercan On, Ali Aytac, Serkan Menekse, Yasemin Bakkal Temi, Kazim Uygun, Elif Sahin, Merve Keskinkilic, Zafer Arik

**Affiliations:** 1 Department of Medical Oncology, Hacettepe University Faculty of Medicine, 06100 Ankara, Türkiye; denizcguven@hotmail.com (D.C.G.); zafer.arik@hacettepe.edu.tr (Z.A.); 2 Department of Medical Oncology, Ege University Faculty of Medicine, 35100 Izmir, Türkiye; hasan-cagri@windowslive.com (H.C.Y.); dr.sercanon@gmail.com (S.O.); 3 Department of Medical Oncology, Baskent University Adana Kisla Hospital, 01120 Adana, Türkiye; fatihkose@gmail.com; 4 Department of Medical Oncology, Izmir City Hospital, 35330 Izmir, Türkiye; grkmkymn@gmail.com (G.K.); ozdemirozlem.md@gmail.com (O.O.); 5 Department of Medical Oncology, Numune Training and Research Hospital, 06230 Ankara, Türkiye; ugurozberk94@gmail.com (U.O.); efnanalgin@gmail.com (E.A.); 6 Cerrahpasa Faculty of Medicine, Department of Medical Oncology, Istanbul University-Cerrahpasa, 34098 Istanbul, Türkiye; drguliyev892@gmail.com (M.G.); nebiserkan.demirci@iuc.edu.tr (N.S.D.); ozkan.alan@hotmail.com (O.A.); 7 Department of Medical Oncology, Aydin Adnan Menderes University Faculty of Medicine, 09100 Aydin, Türkiye; baklaci.ahmet@gmail.com (A.B.); bilgin287@hotmail.com (B.D.); 8 Anadolu Medical Center, Department of Medical Oncology, 41400 Kocaeli, Türkiye; ozlem.topkaya@anadolusaglik.org (O.T.); necdet.uskent@anadolusaglik.org (N.U.); 9 Department of Medical Oncology, Gazi University Faculty of Medicine, 06560 Ankara, Türkiye; kadriyebiryucel@gmail.com (K.B.Y.); orhun.akdogan@gazi.edu.tr (O.A.); 10 Department of Medical Oncology, Koc University Hospital, 34010 Istanbul, Türkiye; bahadirkoylu@hotmail.com (B.K.); fatihsclinicalresearch@yahoo.com (F.S.); 11 Department of Medical Oncology, Memorial Ankara Hospital, 06520 Ankara, Türkiye; irembilgetekin@gmail.com (I.B.); kaanhelvaci.klnk@gmail.com (K.H.); 12 Department of Medical Oncology, Dokuz Eylul University Faculty of Medicine, 35340 Izmir, Türkiye; ahmetunal366@gmail.com (A.U.); huseyinsalih.semiz@deu.edu.tr (H.S.S.); 13 Department of Medical Oncology, Kahramanmaras Sutcu Imam University Faculty of Medicine, 46100 Kahramanmaras, Türkiye; drteomansakalar@gmail.com; 14 Department of Medical Oncology, Marmara University Pendik Training and Research Hospital, 34890 Istanbul, Türkiye; dr.nadya@hotmail.com; 15 Department of Medical Oncology, Pamukkale University Faculty of Medicine, 20070 Denizli, Türkiye; cerenmordag@gmail.com (C.M.C.); ggokoz@pau.edu.tr (G.G.D.); 16 Department of Medical Oncology, Cukurova University Faculty of Medicine, 01330 Adana, Türkiye; sedatb23@hotmail.com (S.B.); ertugrulbayram@cu.edu.tr (E.B.); 17 Department of Medical Oncology, Afyonkarahisar University of Health Sciences, 03200 Afyonkarahisar, Türkiye; drcanansarikayayildiz@gmail.com (C.Y.); hacer.demir@afsu.edu.tr (H.D.); 18 Department of Medical Oncology, Trakya University Faculty of Medicine, 22030 Edirne, Türkiye; dr.ismaiilbayrakci@hotmail.com (I.B.); bulenterdogan@trakya.edu.tr (B.E.); 19 Department of Medical Oncology, MEDDEM Hospital, 32200 Isparta, Türkiye; drkalender@hotmail.com; 20 Department of Medical Oncology, Antalya Training and Research Hospital, 07050 Antalya, Türkiye; hgguzell@gmail.com (H.G.G.); drbanutr@yahoo.com (B.O.); 21 Department of Medical Oncology, Ankara Oncology Training and Research Hospital, 06200 Ankara, Türkiye; drbkarabuga@gmail.com (B.K.); ozturk.ates@hacettepe.edu.tr (O.A.); 22 Department of Medical Oncology, Manisa Celal Bayar University Faculty of Medicine, 45030 Manisa, Türkiye; bihtereniseler@gmail.com (E.B.C.); m_sahbazlar@hotmail.com (M.S.); 23 Department of Medical Oncology, Mersin City Training and Research Hospital, 33090 Mersin, Türkiye; dr.ainal@gmail.com; 24 Department of Medical Oncology, LOSANTE Children and Adult Hospital, 06520 Ankara, Türkiye; velisunar@gmail.com (V.S.); dr.fatmabb@gmail.com (F.B.B.); 25 Department of Medical Oncology, Karadeniz Technical University Faculty of Medicine, 61080 Trabzon, Türkiye; dr.esrakaya@hotmail.com (E.A.); atilayildirim@ktu.edu.tr (A.Y.); 26 Department of Medical Oncology, Ankara University Faculty of Medicine, 06230 Ankara, Türkiye; nejatemreoksuz@gmail.com (N.E.O.); yukselurun@gmail.com (Y.U.); 27 Department of Medical Oncology, VM Medical Park Hospital, Toros University, 33200 Mersin, Türkiye; drerdincnayir@gmail.com; 28 Department of Medical Oncology, VM Medical Park Maltepe Hospital, 34844 Istanbul, Türkiye; sema.turker@yahoo.com; 29 Department of Medical Oncology, Sanliurfa Mehmet Akif Inan Training and Research Hospital, 63050 Sanliurfa, Türkiye; dr_aliaytac@hotmail.com; 30 Department of Medical Oncology, Manisa City Hospital, 45040 Manisa, Türkiye; drsermen@hotmail.com; 31 Department of Medical Oncology, Kocaeli University Faculty of Medicine, 41380 Kocaeli, Türkiye; yasemintemi1@hotmail.com (Y.B.T.); kazim.uygun@kocaeli.edu.tr (K.U.); 32 Department of Medical Oncology, Kocaeli City Hospital, 41090 Kocaeli, Türkiye; 33 Department of Medical Oncology, Izmir Buca Seyfi Demirsoy Training and Research Hospital, 35390 Izmir, Türkiye; mervekeskinkilic90@gmail.com

**Keywords:** ovarian cancer, PARP inhibitors, first-line maintenance, real-world data, BRCA mutation, progression-free survival

## Abstract

**Background:** Poly(ADP-ribose) polymerase (PARP) inhibitors have been established as a first-line maintenance therapy in advanced epithelial ovarian cancer (EOC) following platinum-based chemotherapy. While phase III trials have demonstrated significant progression-free survival (PFS) benefits with olaparib and niraparib, real-world data remain limited. **Methods:** This retrospective, multicenter real-world study included 179 patients with newly diagnosed epithelial ovarian treated with first-line maintenance olaparib or niraparib across 33 centers in Türkiye between January 2014 and March 2025. Clinical, pathological, and molecular data—including BRCA (Breast Cancer Susceptibility Gene) mutation status, origin, and variant classification—was collected. The primary endpoint was PFS, and secondary endpoints included overall survival (OS) and safety. Survival outcomes were analyzed using Kaplan–Meier methods. **Results:** Of 179 patients, 110 received olaparib and 69 received niraparib. BRCA mutations were present in 88.3% of patients, while 11.7% had unknown HRD status. Median follow-up was 16.5 months, and median PFS was not reached. Estimated PFS rates for the overall cohort were 91.0% at 6 months, 83.0% at 12 months, and 64.0% at 24 months. In the olaparib cohort, BRCA-mutant patients demonstrated PFS rates of 89%, 78%, 73%, and 64% at 6, 12, 18, and 24 months, respectively. In the niraparib cohort, corresponding PFS rates among BRCA-mutant patients were 87% at 6 months and 75% at 12 months. Patients harboring pathogenic BRCA variants experienced longer PFS compared with those with likely pathogenic variants. Any-grade adverse events occurred in 73.7% of patients, and grade 3–4 events in 29.6%, with hematologic toxicities predominating. Dose interruptions were more frequent with niraparib, while treatment discontinuation rates were low in both groups. No cases of myelodysplastic syndrome or acute myeloid leukemia were observed. **Conclusions:** In this large multicenter real-world cohort, first-line maintenance therapy with olaparib and niraparib provided durable PFS benefit in patients with advanced EOC, particularly among those with pathogenic BRCA mutations, confirming their effectiveness and manageable safety profiles in routine clinical practice.

## 1. Introduction

Ovarian cancer (OC) remains a significant global health challenge and consistently ranks among the leading causes of gynecological cancer-related mortality [1,2,3,4]. According to GLOBOCAN 2022, there were 324,603 newly diagnosed cases of ovarian cancer worldwide in that year, with 206,956 deaths attributed to the disease in the same year [5].

In epithelial ovarian cancers, 37% of patients are diagnosed at stage III and 28% at stage IV, indicating that the majority present with advanced disease [6]. A major concern associated with advanced OC is its high recurrence rate, which can reach up to 80%, with approximately 70% of patients with stage III–IV high-grade ovarian cancer experiencing relapse within three years [7,8]. Both the advanced stage at diagnosis for most patients and the high recurrence rate, even after optimal surgery and adjuvant chemotherapy, highlight the need for novel strategies to improve outcomes. The standard-of-care first-line treatment for newly diagnosed advanced epithelial ovarian cancer [EOC] typically involves cytoreductive surgery combined with platinum-based chemotherapy [7,8,9,10,11,12].

Following this initial treatment, maintenance therapy has emerged as an increasingly standard approach to reduce recurrence or relapse rates [9,10,13,14,15,16,17,18]. Among the most impactful advances in this setting are poly(ADP-ribose) polymerase (PARP) inhibitors, such as niraparib and olaparib, which have demonstrated significant efficacy in prolonging progression-free survival (PFS) in patients responding to platinum-based chemotherapy. PARP inhibitors exert their antitumor effects by exploiting defects in homologous recombination repair (HRR), triggering synthetic lethality and selectively driving tumor cell death, thereby significantly enhancing treatment efficacy. Key randomized controlled trials (RCTs), including SOLO-1, PRIMA, PRIME, PAOLA-1, and ATHENA-MONO, have firmly established the clinical benefit of PARP inhibitor maintenance therapy [9,10,13,14,15,16,17,18,19,20,21,22,23].

Pivotal randomized controlled trials were conducted in carefully selected and relatively homogeneous patient populations under controlled conditions, with strict eligibility criteria and predefined treatment protocols. Although these trials established the clinical efficacy of PARP inhibitors, their findings may not fully reflect outcomes in routine clinical practice, where patient populations are more heterogeneous with respect to molecular characteristics, comorbidities, performance status, and treatment patterns. Consequently, real-world data are essential to better characterize the effectiveness and safety of PARP inhibitors outside the controlled trial setting.

Despite the increasing use of first-line maintenance PARP inhibitors, large multicenter real-world data remain limited, particularly in middle-income countries. In Türkiye, the effectiveness and safety of first-line maintenance olaparib and niraparib in advanced ovarian cancer have not been evaluated in a nationwide cohort.

To address this gap, we conducted a nationwide multicenter retrospective study to evaluate progression-free survival outcomes and the impact of BRCA mutation status in patients receiving first-line maintenance olaparib or niraparib.

## 2. Materials and Methods

This retrospective, multicenter cohort study included patients from 33 institutions across Türkiye who initiated first-line maintenance therapy with either olaparib or niraparib between 1 January 2014 and 1 March 2025. Survival outcomes were analyzed as of the predefined data cutoff date of 1 March 2025. Patients who experienced disease progression or death before this date were recorded as events, while those alive without progression were administratively censored at the cutoff date. Eligible patients had epithelial ovarian, fallopian tube, or primary peritoneal cancer and underwent either primary cytoreductive surgery or interval debulking following neoadjuvant chemotherapy. Additionally, patients who did not undergo cytoreductive surgery due to medical contraindications or patient preference but received first-line platinum-based chemotherapy followed by PARP inhibitor maintenance were also included. Patients with missing essential clinical data were excluded.

Clinical, demographic, and pathological data, including BRCA mutation status (germline or somatic), were retrospectively retrieved from institutional medical records. The absence of molecular testing in a subset of patients reflected earlier treatment periods during which comprehensive molecular profiling was not yet part of routine clinical practice.

Tumor assessments were performed at baseline and every 12 weeks thereafter using contrast-enhanced computed tomography (CT) or magnetic resonance imaging (MRI). Radiologic responses were assessed by local radiologists, and all determinations of disease progression were confirmed by a central review panel. Central review was performed by experienced radiologists blinded to treatment allocation.

The primary endpoint was progression-free survival (PFS), defined as the interval from completion of platinum-based chemotherapy to the first documentation of radiologic progression, death from any cause, or last follow-up. The secondary endpoint was overall survival (OS), defined as the time from initiation of PARP inhibitor maintenance therapy to death from any cause. Maintenance therapy was continued until radiologic or clinical disease progression. In accordance with guideline recommendations, the planned duration of maintenance therapy was 2 years for olaparib and 3 years for niraparib.

Adverse events were assessed and graded according to the Common Terminology Criteria for Adverse Events (CTCAEs), version 5.0. Dose modifications, temporary interruptions, permanent discontinuations, and laboratory parameters were recorded to evaluate treatment-related toxicity.

All statistical analyses were conducted using IBM SPSS Statistics for Windows, version 25.0. The distribution of variables was assessed using the Kolmogorov–Smirnov and Shapiro–Wilk tests. Normally distributed variables were compared between the olaparib and niraparib groups using Student’s *t*-test, whereas non-normally distributed variables were analyzed using the Mann–Whitney U test. Categorical variables were summarized as frequencies and percentages, and comparisons were performed using Pearson’s chi-square test or Fisher’s exact test, as appropriate. A two-tailed *p*-value < 0.05 was considered statistically significant.

Comparative analyses evaluated potential differences in clinical and demographic characteristics, including age (continuous and categorized as <65 vs. ≥65 years), ECOG performance status (0–1 vs. ≥2), body mass index (BMI; <25 vs. ≥25 kg/m^2^), histologic subtype (serous vs. other), disease stage at diagnosis (early stage, stage III, or stage IV), and disease status at the initiation of PARP inhibitor therapy (non-metastatic vs. metastatic). Molecular characteristics—including BRCA mutation status, mutation origin (somatic vs. germline), BRCA mutation subtype (BRCA1, BRCA2, or BRCA1+2), and variant classification (pathogenic vs. likely pathogenic)—were also compared between treatment groups.

Treatment-related variables included the type of perioperative chemotherapy (adjuvant vs. neoadjuvant), extent of cytoreductive surgery (maximal, optimal, or suboptimal), and serum CA-125 levels measured immediately prior to maintenance therapy, analyzed both as continuous variables and dichotomized at the 35 U/mL threshold. Dose reductions, temporary interruptions, and permanent discontinuations were compared between treatment groups.

The duration of follow-up was estimated using the reverse Kaplan–Meier method. For progression-free survival, events were defined as radiologic/clinical disease progression or death from any cause, whichever occurred first. For overall survival, the event was death from any cause. Patients without an event were censored at the date of last contact, with 1 March 2025 defined as the administrative censoring date.

A Cox proportional hazards model was applied to explore factors associated with PFS. Variables with *p* < 0.2 in univariate analysis and those considered clinically relevant based on prior literature were included in the multivariate model.

Generative artificial intelligence tools were not used in the design, data collection, analysis, or interpretation of the study. During the preparation of the manuscript, ChatGPT (OpenAI, version 5.2) was used solely for language editing, grammar correction, and improvement of clarity. All generated content was carefully reviewed and edited by the authors, who take full responsibility for the integrity, accuracy, and final content of the manuscript.

The multicenter study protocol was reviewed and approved by the institutional Ethics Committee of the coordinating center on 4 March 2025 (Approval No: 2025/06-54). In addition, multicenter ethical approval was obtained in accordance with national regulations, and institutional permissions were secured from all participating centers. The study involved retrospective review of archived medical records. Given the retrospective design and the use of fully anonymized data, individual informed consent was not required in accordance with institutional regulations. The study was conducted in accordance with the principles of the Declaration of Helsinki.

The data supporting the findings of this study are not publicly available due to ethical and privacy restrictions related to patient confidentiality but are available from the corresponding author upon reasonable request.

## 3. Results

### 3.1. Baseline Clinical and Molecular Characteristics

A total of 179 patients with newly diagnosed epithelial ovarian cancer were retrospectively identified across 33 centers in Turkey (see Supplementary Table S1). The median age at initiation of PARP inhibitor therapy was 56.1 years (IQR 48.4–62.8), with 30 patients (16.8%) aged ≥65 years. The cohort’s median body mass index was 26.6 kg/m^2^ (IQR 23.9–29.8), and 115 patients (64.2%) had a BMI >24 kg/m^2^ at treatment initiation. At baseline, 122 patients (68.2%) presented with FIGO stage III disease and 57 (31.8%) with stage IV disease.

Of the 179 patients, 158 (88.3%) harbored BRCA mutations, while HRD status was unknown in 21 patients (11.7%), largely due to limited access to comprehensive HRD testing during earlier treatment periods. Germline or somatic BRCA origin was available for 133 patients (74.3%); among these, 59 (44.4%) had somatic and 74 (55.6%) had germline mutations. Of the 163 patients who underwent cytoreductive surgery, 115 (70.6%) achieved optimal cytoreduction. The best overall response to first-line platinum-based chemotherapy prior to PARP inhibitor maintenance was complete response in 110 patients (61.5%) and partial response in 69 patients (38.5%).

Having characterized the overall cohort, we next compared baseline clinical and molecular features between the olaparib and niraparib treatment groups.

### 3.2. Comparison of Baseline Characteristics Between Treatment Cohorts

There were no significant differences between the olaparib and niraparib cohorts in terms of the patients’ baseline clinical characteristics, including age at diagnosis, age category (<65 vs. ≥65 years), ECOG performance status, body mass index classification, FIGO stage at PARP inhibitor initiation, receipt of neoadjuvant chemotherapy, extent of cytoreductive surgery, histologic subtype, response to first-line chemotherapy, duration of maintenance therapy, or pre-treatment CA-125 levels, whether analyzed as continuous variables or using the 35 U/mL cutoff (Table 1).

Overall, the two treatment cohorts were well balanced with respect to baseline demographic and disease-related characteristics, suggesting comparability for subsequent survival analyses.

Similarly, treatment allocation did not differ according to BRCA mutation status (mutant vs. HRD status unknown), mutation origin (somatic vs. germline), or BRCA subtype (BRCA1, BRCA2, or combined BRCA1+2).

In contrast, among the 126 patients with available BRCA variant classification, a significant difference in variant distribution was observed between treatment cohorts (*p* = 0.014). In the olaparib group, 60 patients (76.9%) harbored pathogenic variants and 18 (23.1%) had likely pathogenic variants, whereas in the niraparib cohort, 45 patients (93.8%) carried pathogenic variants and only three (6.3%) were classified as likely pathogenic.

Beyond baseline comparability, we also examined treatment tolerability between cohorts (Table 2). Dose modifications occurred in 30 of 110 patients (27.3%) treated with olaparib and in 27 of 69 patients (39.1%) treated with niraparib, with no statistically significant difference between the two groups (*p* = 0.097). Discontinuations due to adverse events were infrequent and comparable between treatment arms (2/110 (1.8%) vs. 4/69 (5.8%); *p* = 0.150). In contrast, dose interruptions were significantly more common in the niraparib cohort than in the olaparib cohort (21/69 (30.4%) vs. 17/110 (15.5%); *p* = 0.017).

### 3.3. Follow-Up Duration and Survival Outcomes

Following assessment of baseline characteristics and treatment patterns, we evaluated follow-up duration and survival outcomes. As of the data cutoff on 1 March 2025, only eight overall survival (OS) events (4.5% of 179 patients) had occurred, resulting in limited data maturity and precluding meaningful estimation of median OS, which therefore remains unreached. Consequently, OS outcomes could not be robustly evaluated at this time. In addition, disease progression was observed in 39 patients (21.8%), rendering median progression-free survival (PFS) non-estimable.

Using the reverse Kaplan–Meier method, the median follow-up duration for the entire cohort was 16.5 months (95% CI, 15.2–17.8). Median PFS was not reached, as the Kaplan–Meier survival curve did not cross 50% during follow-up; therefore, the 95% confidence interval was not estimable. We noted estimated PFS rates of 91.0% at 6 months, 83.0% at 12 months, 69.0% at 18 months, 64.0% at 24 months, and 60.0% at 30 months.

### 3.4. First-Line Olaparib Outcomes

In the olaparib-treated cohort (n = 110), four patients died and 23 experienced disease progression during follow-up. The median follow-up duration was 12.55 months (95% CI, 10.68–14.42). Overall, 97 patients (88.2%) harbored BRCA mutations, while HRD status was unknown in 13 patients (11.8%).

Among BRCA-mutant patients, cumulative progression-free survival (PFS) rates were 89% at 6 months, 78% at 12 months, 73% at 18 months, and 64% at 24 months. In contrast, patients with unknown HRD status exhibited lower PFS rates of 85% at 6 months, 34% at 12 months, and 23% at 18 months.

Germline or somatic BRCA origin was available for 84 patients (76.4%). Of these, 46 patients (54.8%) had germline mutations and 38 (45.2%) had somatic mutations. Mean PFS was numerically longer in patients with germline mutations compared with those with somatic mutations (35.3 vs. 22.5 months), although this difference did not reach statistical significance (*p* = 0.088).

At the initiation of PARP inhibitor therapy, 79 patients (71.8%) had stage III disease and 31 (28.2%) had stage IV disease, with no significant difference in PFS between stages. Among the 101 patients who underwent surgery, 41 (40.6%) received neoadjuvant chemotherapy and 60 (59.4%) underwent upfront surgery; surgical approach was not associated with PFS.

Patients achieving a complete response (CR) after first-line platinum-based chemotherapy demonstrated significantly longer mean PFS compared with those achieving a partial response (PR) (31.9 vs. 22.5 months; *p* = 0.014). To further explore factors associated with PFS, we performed a univariate Cox regression analysis, the results of which are presented in Table 3.

### 3.5. First-Line Niraparib Outcomes

In the niraparib-treated cohort (n = 69), 61 patients (88.4%) harbored BRCA mutations, while HRD status was unknown in eight patients (11.6%). During follow-up, 16 patients experienced disease progression and 4 patients died. The median follow-up duration was 12.55 months (95% CI, 10.68–14.42).

Among BRCA-mutant patients, the estimated progression-free survival (PFS) rates were 87% at 6 months and 75% at 12 months. In contrast, patients with unknown HRD status demonstrated PFS rates of 82% at 6 months, which declined to 41% at 12 months.

Information on BRCA mutation origin (somatic vs. germline) was available for 49 patients; 21 (42.9%) had somatic mutations and 28 (57.1%) had germline mutations. The mean PFS was 41.8 months in the somatic group and 36.4 months in the germline group, with no statistically significant difference observed (*p* = 0.999).

At the initiation of PARP inhibitor therapy, 43 patients had stage III disease and 26 had stage IV disease. Mean PFS was significantly longer in patients with stage III disease compared with those with stage IV disease (43.2 vs. 15.6 months; *p* = 0.015).

Of the 69 patients, 62 underwent cytoreductive surgery, including 26 (41.9%) who had upfront surgery and 36 (58.1%) who received neoadjuvant chemotherapy. Patients who underwent upfront surgery had a significantly longer mean PFS compared with those receiving neoadjuvant chemotherapy (48.4 vs. 36.5 months; *p* = 0.021).

According to response to first-line platinum-based chemotherapy, 39 patients achieved a complete response (CR) and 30 achieved a partial response (PR). Mean PFS was longer in the CR group than in the PR group (38.1 vs. 24.2 months); however, this difference did not reach statistical significance (*p* = 0.901). The results of the Cox regression analysis for PFS in patients receiving first-line niraparib are presented in Table 4.

All other evaluated clinical and treatment-related variables—including age, BMI, extent of debulking, residual disease, and treatment modifications due to toxicity—were individually assessed and showed no significant association with PFS in either the olaparib- or niraparib-treated cohorts.

Across the entire study population (n = 179), ECOG performance status ≥1 emerged as an independent predictor of PFS and was associated with worse outcomes (HR = 0.431; 95% CI, 0.199–0.934; *p* = 0.033). No other evaluated covariates showed an independent association with PFS.

Collectively, these findings suggest broadly comparable real-world effectiveness between first-line olaparib and niraparib, with specific prognostic factors differing across treatment cohorts.

### 3.6. Safety and Treatment-Related Adverse Events

In addition to efficacy outcomes, safety and treatment-related adverse events were systematically evaluated. Any-grade adverse events were reported in 132 of 179 patients (73.7%), while grade 3–4 events occurred in 53 patients (29.6%). No secondary malignancies, including acute myeloid leukemia or myelodysplastic syndromes, were observed during follow-up.

In the olaparib cohort, any-grade adverse events occurred in 78 of 110 patients (70.9%), and grade 3–4 events in 29 patients (26.4%). The most frequent grade ≥3 hematologic adverse event was anemia (72.4%), followed by thrombocytopenia (6.9%) and neutropenia (6.9%). Among non-hematologic toxicities, grade 3–4 fatigue was the most common (31.0%). Dose interruption, dose reduction, and treatment discontinuation occurred in 15.5%, 27.3%, and 1.8% of patients, respectively.

In the niraparib cohort, any-grade adverse events were reported in 54 of 69 patients (78.3%), with grade 3–4 events occurring in 24 patients (34.8%). The most frequent hematologic adverse event grade ≥3 was anemia (54.2%), followed by thrombocytopenia (29.2%) and neutropenia (16.7%). Grade 3–4 non-hematologic adverse events were less frequent, with nausea reported in 12.5% and fatigue in 8.3% of patients. Dose interruption was more common in this cohort (30.4%), while dose reduction and treatment discontinuation occurred in 39.1% and 5.8% of patients, respectively.

## 4. Discussion

This nationwide multicenter study provides the first large-scale comparative real-world analysis of first-line olaparib and niraparib maintenance therapy in advanced ovarian cancer in Türkiye.

In our niraparib cohort, the estimated PFS rates among patients with BRCA mutations were 87% at 6 months and 75% at 12 months. Compared with the PRIMA trial, in which BRCA-mutant patients receiving niraparib achieved PFS rates of 98% and 77.6% at 6 and 12 months, respectively, our findings indicate comparable efficacy at one year, despite a slightly higher rate of early progression within the first 6 months [24].

In our olaparib cohort, BRCA-mutant cases (n = 97) demonstrated cumulative PFS rates of 89%, 78%, 73%, and 64% at 6, 12, 18, and 24 months, respectively. Compared with the SOLO1 trial, where corresponding PFS rates were 92.3%, 88.1%, 85.0%, and 81.5%, our results indicate comparable short-term outcomes but a modest attenuation of PFS beyond 12 months in the real-world setting [20].

In our study, patients who achieved a complete response (CR) after chemotherapy had a significantly longer mean PFS compared with those with a partial response (PR) (31.9 vs. 22.5 months, *p* = 0.014). This finding is consistent with real-world series and supports the notion that the depth of initial response to first-line chemotherapy is an important determinant of progression-free survival under PARP inhibitor maintenance therapy [25,26,27].

In our study, no significant difference in PFS was observed between patients who underwent upfront surgery and those who received neoadjuvant chemotherapy (NACT). Consistently, the SOLO1 subgroup analysis demonstrated that the PFS benefit of olaparib was maintained irrespective of prior NACT exposure, suggesting that surgical timing does not influence the efficacy of PARP inhibition [20].

In our cohort, patients with stage III disease had a significantly longer PFS than those with stage IV disease (43.2 vs. 15.6 months, *p* = 0.015). This finding aligns with the PRIMA trial, in which the higher proportion of stage IV patients reflected a worse prognosis, and stage IV disease was associated with shorter survival compared with stage III disease (HR 1.40 vs. 0.86] [24].

In the niraparib cohort, patients who achieved a complete response (CR) had a numerically longer mean PFS compared with those who achieved a partial response (PR) (38.1 vs. 24.2 months), although this difference did not reach statistical significance (*p* = 0.901). Similarly, in the PRIMA trial, median PFS was longer among patients with CR (16.4 months) than among those with PR (8.3 months) [24]. A Chinese real-world analysis of PARP inhibitors also reported a trend toward longer PFS in CR patients; however, statistical significance was not achieved, which was attributed to immature follow-up data [28]. Taken together, these findings suggest a consistent numerical trend favoring CR, indicating that depth of response to first-line chemotherapy may influence PFS outcomes under niraparib maintenance, although larger cohorts and longer follow-up are required to confirm this association.

In the niraparib cohort, patients who underwent upfront surgery had a significantly longer PFS compared with those who received NACT (48.4 vs. 36.5 months, *p* = 0.021). By contrast, the PRIMA trial demonstrated that niraparib provided a similar PFS benefit regardless of NACT exposure (HR 0.59 for NACT vs. HR 0.67 for no NACT), indicating that treatment effect was independent of surgical timing. This discrepancy may be partially explained by differences in patient selection, baseline disease burden, surgical outcomes, or sample size limitations in our cohort [24].

Regarding safety, in the olaparib cohort, any-grade adverse events occurred in 70.9% of patients, with grade 3–4 events observed in 26.4%. Hematologic toxicities predominated, particularly anemia (72.4%), followed by thrombocytopenia (6.9%) and neutropenia (6.9%), while grade 3–4 fatigue (31.0%) was the most frequent non-hematologic adverse event. In the SOLO1 trial, any-grade adverse events were reported in nearly all patients (98.5%), with grade ≥3 events occurring in 39.6%, most commonly anemia (21.9%) and neutropenia (8.5%) [20]. Despite the higher proportion of grade ≥3 anemia in our cohort, rates of treatment discontinuation (1.8%) and dose interruption (15.5%) were lower than those reported in SOLO1, suggesting effective toxicity management in routine clinical practice.

In the niraparib cohort, treatment-emergent adverse events were observed in 78.3% of patients, with grade 3–4 events occurring in 34.8%. Hematologic toxicities were the most common, including anemia (54.2%), thrombocytopenia (29.2%), and neutropenia (16.7%), whereas severe non-hematologic adverse events were infrequent. Compared with PRIMA (any grade 99%, grade ≥3 72.9%) and other real-world series, our cohort demonstrated a lower overall burden of severe toxicity and a low discontinuation rate (5.8%), likely reflecting effective dose interruption and reduction strategies [24,29].

No cases of myelodysplastic syndrome or acute myeloid leukemia were observed; however, the follow-up duration was insufficient to exclude these rare late toxicities.

The strengths of this study include its relatively large real-world cohort and one of the most detailed comparative evaluations of olaparib and niraparib in the first-line maintenance setting. Unlike most previous studies, which did not analyze germline and somatic BRCA subgroups separately, our study incorporated these distinctions along with key clinical variables such as surgical outcomes, BMI, age, and treatment timing, providing a more nuanced understanding of treatment response. The consistency of our results with pivotal trials and large real-world datasets further supports the reliability and external validity of our findings.

This study has several limitations. The relatively short median follow-up (12.95 months) limits robust evaluation of overall survival, late toxicities, and long-term PFS patterns. The low number of progression events and high censoring in certain subgroups may have reduced statistical power and generalizability. The absence of independent predictors in the olaparib cohort and the limited significant variables in the niraparib cohort likely reflect these power constraints, and modest associations may have remained undetected. Given its multicenter retrospective design, the study may be subject to inter-center variability in clinical practice and patient management, as well as selection bias and missing data, which should be considered when interpreting the findings.

Future prospective real-world studies incorporating comprehensive HRD profiling and longer follow-up are warranted to better define patient subgroups most likely to derive sustained benefit from first-line PARP inhibitor maintenance.

## 5. Conclusions

These nationwide real-world data suggest that both olaparib and niraparib are feasible and effective options for first-line maintenance therapy in newly diagnosed advanced ovarian cancer in routine practice. In our cohort, pathogenic BRCA variant status was associated with prolonged progression-free survival and remained a key prognostic factor in multivariable analyses. These findings highlight the clinical importance of timely and comprehensive genetic testing and support biomarker-based risk stratification when interpreting outcomes in the first-line maintenance setting. Given the retrospective design and limited event maturity, particularly for overall survival, further prospective studies and longer follow-up are warranted to confirm comparative effectiveness and long-term safety.

## Figures and Tables

**Table 1:** Baseline clinical and molecular characteristics.

Clinical Characteristics	Olaparib (n = 110)	Niraparib (n = 69)	*p*-Value
Age (mean ± SD). Years	55.4 ± 10.3	56.1 ± 9.8	0.62
<64 (n. %)	89 (80.9%)	60 (87%)	0.31
≥65 (n. %)	21 (19.1%)	9 (13%)	
ECOG performance status (n%)			0.35
0	59 (53.6%)	42 (60.9%)	
1–2	51 (46.4%)	27 (39.1%)	
BMI			0.13
Normal <25	44 (40%)	20 (29%)	
Overweight	66 (60%)	49 (71%)	
Stage at PARPi initiation			0.19
Stage 3	79 (71.8%)	43 (62.3%)	
Stage 4	31 (28.2%)	26 (37.7%)	
HRD status (n%)			0.81
Deficient	97 (88.2%)	62 (89.9%)	
Unknown	13 (11.8%)	7 (10.1%)	
BRCA gene (n%)			0.96
Mutant	97 (88.2%)	61 (88.4%)	
Unknown	13 (11.8%)	8 (11.6%)	
Mutation origin (n%)			
Somatic	38 (45.2%)	25 (47.2%)	0.82
Germline	51 (60.7%)	32 (60.4%)	0.96
BRCA mutation subtype (n. %)			0.69
BRCA 1	59 (62.8%)	32 (60.4%)	
BRCA 1/2	8 (8.5%)	3 (5.7%)	
BRCA 2	27 (28.7%)	18 (34.0%)	
BRCA Variant Classification			0.014
Pathogenic	60 (76.9%)	45 (93.8%)	
Likely Pathogenic	18 (23.1%)	3 (6.3%)	
Primary treatment			0.08
Adjuvant	60 (54.5%)	26 (37.7%)	
Neoadjuvant	41 (37.3%)	36 (52.2%)	
Non-operative	9 (8.2%)	7 (10.1%)	
Surgery type			0.22
No surgery	9 (8.2%)	7 (10.1%)	
Maximal	55 (50.0%)	28 (40.6%)	
Optimal	22 (20.0%)	10 (14.5%)	
Suboptimal	24 (21.8%)	24 (34.8%)	
Response to first-line chemotherapy.			0.28
CR	71 (64.5%)	39 (56.5%)	
PR	39 (35.5%)	30 (43.5%)	
CA125 before CT	361 (IQR 92–1240)	759 (IQR 136–1474)	0.14
CA125 before PARPi	9.6 (IQR 6.7–17.3)	10.5 (IQR 6.9–18)	0.40
CA125 before PARPi			0.60
≤35 U/mL	94 (90.4%)	58 (87.9%)	
>35 U/mL	10 (9.6%)	8 (12.1%)	
Duration of PARPi. median (Q1–Q3) mo	10.17 (6.9–16.6)	10.22 (6.4–14.9)	0.46

**Table 2:** Treatment modifications according to PARP inhibitor type.

Adverse Events	Olaparib (n = 110)	Niraparib (n = 69)	*p*-Value
Dose modification (n%)	30 (27.3%)	27 (39.1%)	0.09
Dose interruption (n%)	17 (15.5%)	21 (30.4%)	0.01
Dose discontinuation (n%)	2 (1.8%)	4 (5.8%)	0.15

**Table 3:** Regression analysis for PFS in patients receiving first-line olaparib *.

	Univariate HR (95% CI)	*p*-Value
Age (<65 vs. ≥65)	0.59 (0.17–2.09)	0.42
BMI (Normal vs. Overweight)	0.78 (0.33–1.85)	0.58
Debulking: Optimal vs. Maximal	1.02 (0.35–2.92)	0.96
Debulking: Suboptimal vs. Maximal	0.46 (0.10–1.99)	0.30
Residual disease (Y/N)	0.83 (0.29–2.31)	0.72
Stage (non-metastatic vs. metastatic)	1.20 (0.47–3.04)	0.70
NACT + Surgery (Y/N)	1.10 (0.43–2.78)	0.83
PARPi interruption (Y/N)	1.56 (0.46–5.30)	0.47
PARPi dose reduction (Y/N)	0.94 (0.38–2.31)	0.90
BRCA germline	2.10 (0.67–6.58)	0.20
BRCA somatic	2.65 (0.83–8.49)	0.10
BRCA Variant Classification	0.30 (0.09–1.02)	0.05

* Variables with a *p* value < 0.20 in univariate analysis were entered into the multivariate Cox regression model; however, no variables were identified as independent predictors of progression-free survival.

**Table 4:** Cox regression analysis for PFS in patients receiving first-line niraparib *.

	Univariate HR (95% CI)	*p*-Value	Multivariate HR (95% CI)	*p*-Value
Age (<65/≥65)	0.46 (0.14–1.44)	0.18		
BMI (Normal/ Overweight)	0.31 (0.07–1.38)	0.12		
Debulking: Optimal/Maximal	0.60 (0.16–2.26)	0.46		
Debulking: Suboptimal/Maximal	0.41 (0.04–3.54)	0.42		
Residual disease (Yes/No)	0.55 (0.16–1.92)	0.35		
BRCA status (Mutated/Unknown)	0.43 (0.14–1.35)	0.15		
Stage (non-metastatic/metastatic)	0.30 (0.11–0.83)	0.02		
NACT + Surgery (Yes/No)	0.12 (0.01–1.00)	0.05		
PARPi interruption due to toxicity (Yes/No)	0.53 (0.20–1.44)	0.22		
PARPi dose reduction due to toxicity (Yes/No)	0.74 (0.27–2.03)	0.56		
BRCA germline	1.54 (0.40–5.92)	0.52		
BRCA somatic	1.06 (0.32–3.54)	0.91		
BRCA variant (pathogenic/likely)	0.09 (0.01–0.53)	<0.01	0.02 (0.00–0.39)	0.01

* Variables with *p* < 0.20 in univariate analysis were entered into the multivariate Cox regression model. In multivariate analysis, BRCA variant classification remained the only independent predictor of PFS (HR 0.021, 95% CI 0.001–0.395; *p* = 0.010).

## Data Availability

The data presented in this study are not publicly available due to ethical and privacy restrictions. Data are available from the corresponding author upon reasonable request.

## Supplementary Materials

The following supporting information can be downloaded at: https://www.mdpi.com/article/10.3390/jcm15041657/s1, Table S1: Distribution of Patients Across Participating Centers (n = 179).

## Abbreviations

The following abbreviations are used in this manuscript:

AE	Adverse Event
AML	Acute Myeloid Leukemia
BRCA	Breast Cancer Susceptibility Gene
CR	Complete Response
EOC	Epithelial Ovarian Cancer
HR	Hazard Ratio
HRD	Homologous Recombination Deficiency
MDS	Myelodysplastic Syndrome
NACT	Neoadjuvant Chemotherapy
PARP	Poly(ADP-ribose) Polymerase
PFS	Progression-Free Survival
PR	Partial Response
TEAE	Treatment-Emergent Adverse Event
