# Supplementary material for: Outcomes of First-Line PARP Inhibitor Therapy in Ovarian Cancer: A Multicenter Retrospective Analysis
Source: J Clin Med. 2026 Feb 22;15(4):1657. doi: 10.3390/jcm15041657 (PMC12942131; doi:10.3390/jcm15041657)
Supplement: Supplementary file 1 [file jcm-15-01657-s001.zip › jcm-4158929-supplementary.pdf]

Supplementary Table S1. Distribution of Patients Across Participating Centers (N = 179)

Rank	Center	Number of Patients
1	Hacettepe University Faculty of Medicine, Department of Medical Oncology, Ankara, Türkiye	21
2	Baskent University Adana Kisla Hospital, Department of Medical Oncology, Adana, Türkiye	16
3	Izmir City Hospital, Department of Medical Oncology, Izmir, Türkiye	11
4	Numune Training and Research Hospital, Department of Medical Oncology, Ankara, Türkiye	10
5	Cerrahpasa Faculty of Medicine, Istanbul University–Cerrahpasa, Department of Medical Oncology, Istanbul, Türkiye	9
6	Aydin Adnan Menderes University Faculty of Medicine, Department of Medical Oncology, Aydin, Türkiye	9
7	Anadolu Medical Center, Department of Medical Oncology, Kocaeli, Türkiye	8
8	Gazi University Faculty of Medicine, Department of Medical Oncology, Ankara, Türkiye	8
9	Koc University Hospital, Department of Medical Oncology, Istanbul, Türkiye	7
10	Memorial Ankara Hospital, Department of Medical Oncology, Ankara, Türkiye	7
11	Marmara University Pendik Training and Research Hospital, Department of Medical Oncology, Istanbul, Türkiye	6
12	Dokuz Eylul University Faculty of Medicine, Department of Medical Oncology, Izmir, Türkiye	6
13	Kahramanmaras Sutcu Imam University Faculty of Medicine, Department of Medical Oncology, Kahramanmaras, Türkiye	6
14	Pamukkale University Faculty of Medicine, Department of Medical Oncology, Denizli, Türkiye	5
15	Cukurova University Faculty of Medicine, Department of Medical Oncology, Adana, Türkiye	5
16	Afyonkarahisar University of Health Sciences, Department of Medical Oncology, Afyonkarahisar, Türkiye	5
17	Ege University Faculty of Medicine, Department of Medical Oncology, Izmir, Türkiye	5
18	Antalya Training and Research Hospital, Department of Medical Oncology, Antalya, Türkiye	4
19	MEDDEM Hospital, Department of Medical Oncology, Isparta, Türkiye	4
20	Trakya University Faculty of Medicine, Department of Medical Oncology, Edirne, Türkiye	4
21	Manisa Celal Bayar University Faculty of Medicine, Department of Medical Oncology, Manisa, Türkiye	3
22	Mersin City Training and Research Hospital, Department of Medical Oncology, Mersin, Türkiye	3
23	Ankara Oncology Training and Research Hospital, Department of Medical Oncology, Ankara, Türkiye	3

24	Karadeniz Technical University Faculty of Medicine, Department of Medical Oncology, Trabzon, Türkiye	2
25	LOSANTE Children and Adult Hospital, Department of Medical Oncology, Ankara, Türkiye	2
26	Ankara University Faculty of Medicine, Department of Medical Oncology, Ankara, Türkiye	2
27	Toros University / VM Medical Park Hospital, Department of Medical Oncology, Mersin, Türkiye	2
28	Sanliurfa Mehmet Akif Inan Training and Research Hospital, Department of Medical Oncology, Sanliurfa, Türkiye	1
29	Manisa City Hospital, Department of Medical Oncology, Manisa, Türkiye	1
30	Kocaeli University Faculty of Medicine, Department of Medical Oncology, Izmit, Kocaeli, Türkiye	1
31	Kocaeli City Hospital, Department of Medical Oncology, Izmit, Kocaeli, Türkiye	1
32	VM Medical Park Maltepe Hospital, Department of Medical Oncology, Istanbul, Türkiye	1
33	Izmir Buca Seyfi Demirsoy Training and Research Hospital, Department of Medical Oncology, Buca, Izmir, Türkiye	1
